# Hybrid Plasmas for Materials Processing

**DOI:** 10.3390/ma16114013

**Published:** 2023-05-27

**Authors:** Reinosuke Kusano, Yukihiro Kusano

**Affiliations:** 1 School of Physics and Astronomy, University of St Andrews, St Andrews KY16 9SS, UK; rk77@st-andrews.ac.uk; 2 Danish Technological Institute, 2630 Taastrup, Denmark

**Keywords:** gas discharge plasma, hybrid plasma, inductively coupled plasma, capacitively coupled plasma, microwave plasma, arc, gliding arc

## Abstract

Hybrid plasmas have been reported in various areas of research over the last 40 years. However, a general overview of hybrid plasmas has never been presented or reported. In the present work, a survey of the literature and patents is carried out to provide the reader with a broad view of hybrid plasmas. The term refers to several different configurations of plasmas, including but not limited to: plasmas driven by several power sources simultaneously or sequentially, plasmas that have the properties of both thermal and nonthermal plasmas, plasmas that are enhanced by additional energy, and plasmas that are operated in a unique medium. In addition, a way of evaluating hybrid plasmas in terms of the improvement of processes is discussed, as well as the negative impacts that follow the employment of hybrid plasmas. Regardless of what the hybrid plasma in question is composed of, it often poses a unique advantage to its nonhybrid counterpart, whether it be used for welding, surface treatment, materials synthesis, coating deposition, gas phase reactions, or medicine.

## 1. Introduction

Gas discharge plasmas are widely applied for materials processing due to their unique properties of reactivities, effectiveness, controllability, and environmental friendliness [1]. For example, plasmas can be applied in the following areas or industries: semiconductors, electronics, automotives, polymers, food, construction, mechanics, medicine, combustion, and energy. With regards to materials processing, plasmas can be used to functionalize surfaces of bulk materials and films as well as synthesize particles. In fact, it is difficult to find industrial areas in which plasmas are not used; plasmas are very useful since they can be used to process solids (bulk, materials, thin films, particles, etc.), liquids, and gases. There have been various attempts to further improve the properties of plasmas, as well as attempts that seek to discover and explore unknown effects. 

The development of hybrid plasma is stimulated by these ventures [2], often operated at atmospheric pressure [3,4]. However, the technical term “hybrid plasma” has been used for several different types of technologies. The present paper reviews different types of hybrid plasmas for materials processing and discusses the scope of future developments. The term “hybrid plasma” can refer to a plasma driven by multiple different sources, a plasma having multiple unique properties simultaneously, or a plasma enhanced by additional energy such as photoirradiation, acoustic energy, or thermal energy. In addition, plasma operated in a unique medium rather than a gaseous phase can also be classified as a hybrid plasma. 

Since the major motive of developing hybrid plasmas is to exhibit something that cannot be achieved by conventional plasma alone, it is natural that these plasmas are often associated with intellectual properties. There are many patents associated with hybrid plasmas filed and granted worldwide for decades. Due to the nature of intellectual properties, operating conditions are ambitiously defined. For example, it is often the case that hybrid plasmas described in patents are described to be operational at both low and atmospheric pressures, even if this is sometimes unrealistic; the hybrid plasmas in these patents do not necessarily demonstrate the proposed effects at both pressures. As a result, their descriptions can sometimes be inaccurate or incorrect. Nonetheless, useful information can be included, and thus, these patents should neither be underestimated nor neglected. 

In the current paper, the following types of hybrid plasmas from academic publications and patents will be presented:

- Plasmas driven by multiple different electrical sources simultaneously;
- Plasmas driven by multiple different electrical sources sequentially;
- Plasmas having properties of thermal and nonthermal plasmas;
- Plasmas enhanced by additional energy;
- Plasmas operated in a unique medium.

Furthermore, proposals and suggestions are made for the future of hybrid plasmas, and the advantages and disadvantages of hybrid plasmas are discussed. 

## 2. Plasmas Driven by Multiple Different Electrical Sources Simultaneously

### 2.1. Combination of Two Plasmas

The technical term “hybrid plasma” was mentioned as early as 1983 [2], and this work by Yoshida et al. can therefore be counted among the earliest works to study hybrid plasmas. This hybrid plasma is described as the superposition of a radio-frequency (RF) plasma and an arc jet. The numerical model of the hybrid plasma predicts its higher efficiency than conventional plasmas. The experimental investigation based on the numerical model exhibits that the hybrid plasma enables the effective synthesis of ultrafine SiNx compounds [2]. 

Similarly, a mathematical model that simulates a plasma reactor, which combines direct current (DC) and RF plasmas for the production of silicon, finds that by coupling flow and temperature fields, both production and recovery of silicon become remarkably more efficient than for a purely DC or RF plasma reactor [5]. 

**Figure 1:**
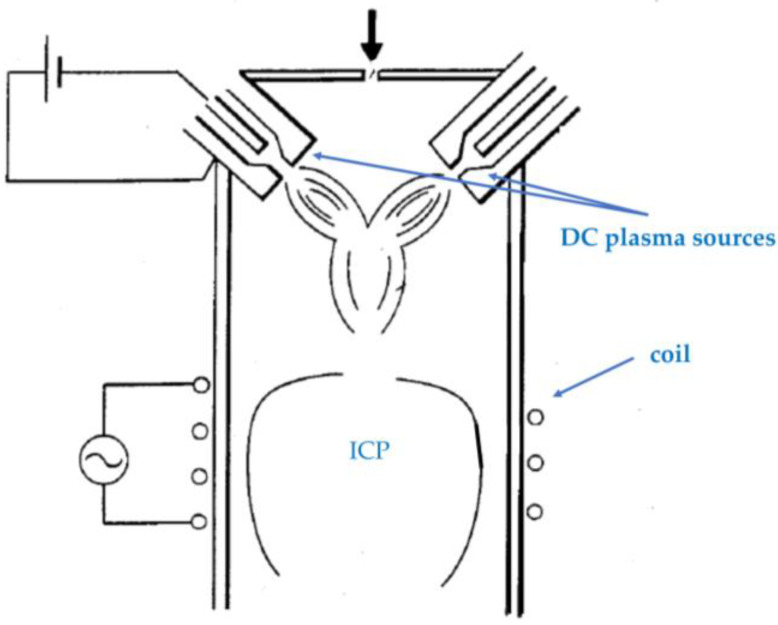
A schematic diagram of an apparatus comprised of a plurality of DC plasma sources with ICP (based on [6]).

Saiki et al. propose a process for producing ultrafine metallic or metal compound particles using an apparatus comprised of a plurality of DC plasma sources with inductively coupled plasma (ICP) [6], as shown in Figure 1. Here, the DC plasma sources generate arc discharges to heat up the source material to synthesize the particles. The ICP is used to induce reactions of the synthesized particles with the surrounding reactive gas. 

**Figure 2:**
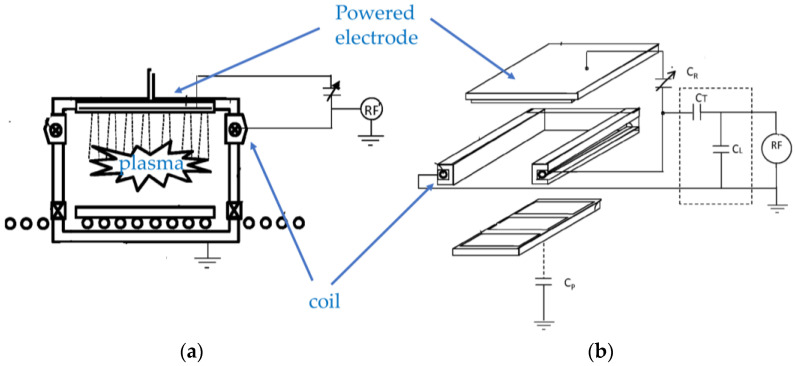
Schematic diagrams of a low-pressure plasma setup. (**a**) A cross-sectional view of the setup; (**b**) A perspective view of the electrodes and coil configuration of the setup with RF generator and matching circuit (based on [7]).

Both capacitively coupled plasma (CCP) and ICP can be generated using RF power supplies. Cho et al. propose a low-pressure plasma setup containing both operated by one generator, as shown in Figure 2 [7]. ICP can be generated in a space between powered and ground electrodes that constitute CCP. The proposed setup allows for high throughput productions for plasma etching and any other general plasma processing due to its simple design. 

**Figure 3:**
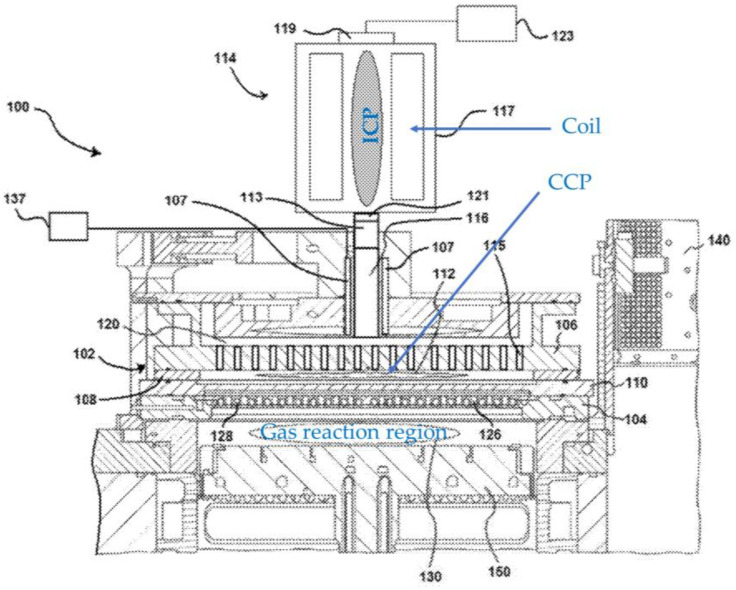
A schematic diagram of a hybrid plasma setup comprised of a remote ICP and CCP (based on [8,9,10]).

Chen et al. [8,9] and Cui et al. [10] also propose the combination of ICP and CCP for semiconductor processing, as shown in Figure 3. The difference from [7] is that ICP is a remote plasma source. The plasma excited species generated at CCP with the aid of ICP is fed to the gas reaction region. A specimen is placed at the gas reaction region to be processed. When higher power operation is required, both plasmas can be used simultaneously so that CCP can be operated at lower power. This configuration can reduce the generation of unwanted contaminations from CCP electrodes. 

**Figure 4:**
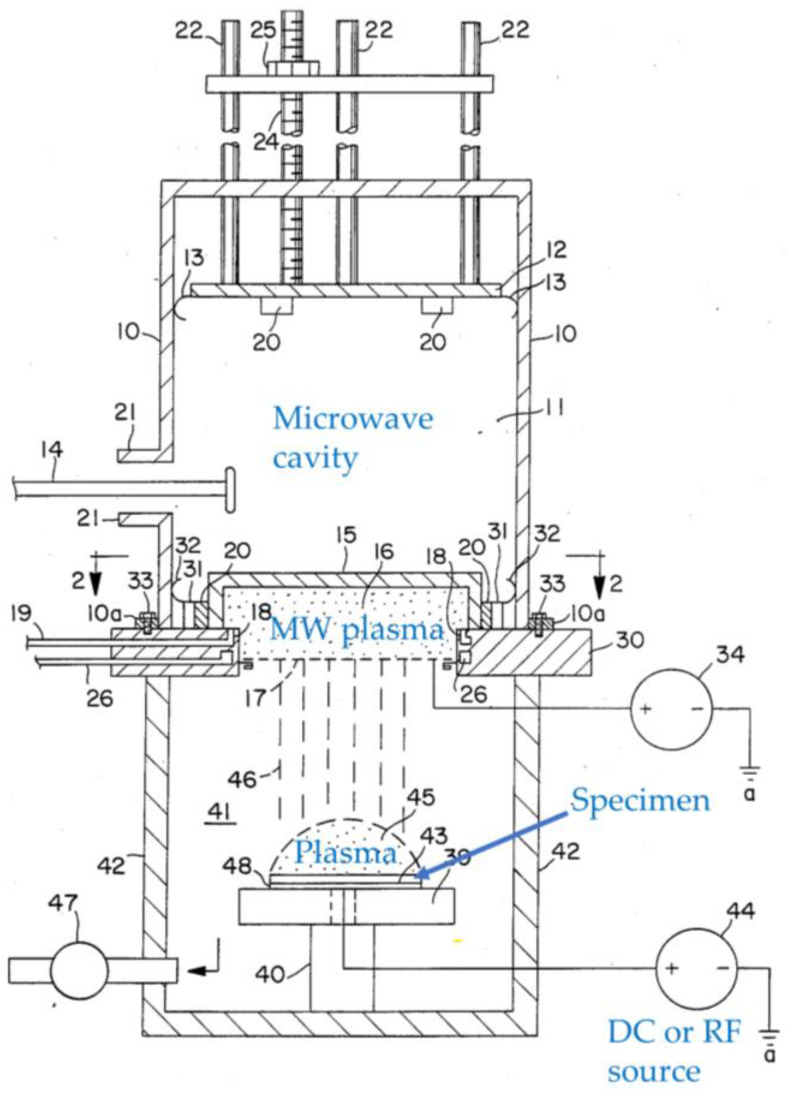
A cross-sectional view of a hybrid plasma setup, where the lower part is a hybrid of MW plasma and DC or RF plasma depending on the way it is biased (based on [11]).

Roppel et al. [11] propose a dual plasma microwave (MW) apparatus. It is a combination of DC or RF plasma with MW, as shown in Figure 4. A DC or RF plasma is generated by supplying DC or RF power to a platform on which a specimen to be treated is placed. A DC or RF plasma is generated above the specimen. Meanwhile, MW is introduced to generate MW plasma. By applying a bias, ions in the MW plasma can be extracted to interact with the DC or RF plasma. This configuration enables pronounced ignition of the DC or RF plasma as well as tuning and improving plasma treatment effects. 

MW plasma and low-frequency (LF) plasma can be used in combination for plasma sterilization at atmospheric pressure [12]. It is discovered that the sterilization effects on spore-forming bacteria depend on the way the plasma gases are supplied [12].

**Figure 5:**
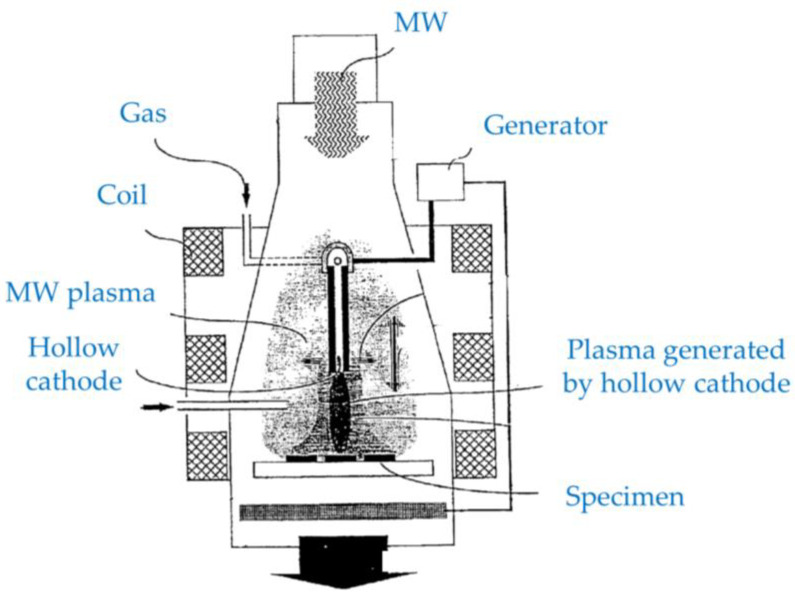
A schematic diagram of a hybrid plasma setup comprised of a plasma generated by a hollow cathode and MW plasma in the presence of a magnetic field (based on [13]).

Bárdoš et al. [13,14] propose a hybrid plasma configuration applicable for low-pressure processing (below 1 Pa) and for the generation of cold atmospheric pressure plasma, which can be used for thin film deposition or plasma surface treatment. The low-pressure hybrid plasma source combines an electron cyclotron resonance (ECR) plasma and a plasma generated by a hollow cathode, as shown in Figure 5. In the case of atmospheric pressure operation, efficient ECR is not expected due to significant collisions of electrons in the plasma. The plasma from the hollow cathode and MW plasma can be simultaneously generated to exhibit high plasma densities without the need for a magnetic field. 

**Figure 6:**
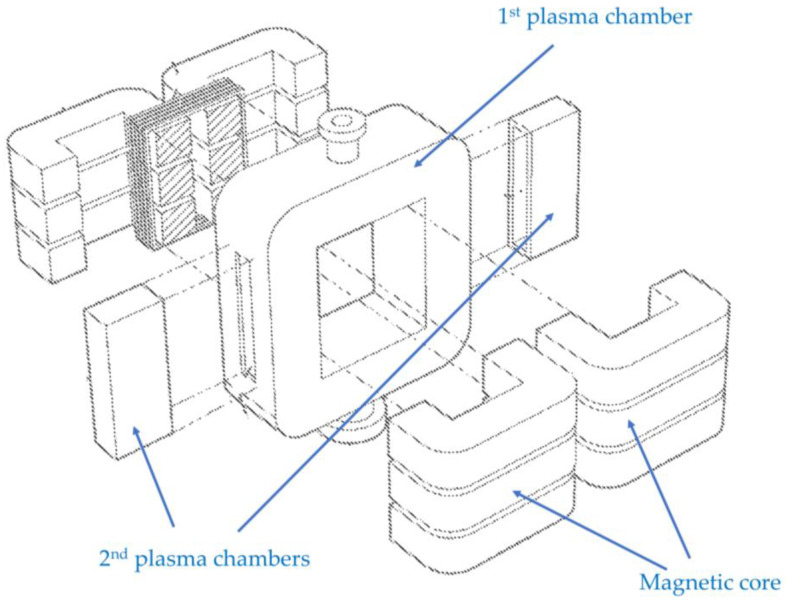
A sectional view of a hybrid plasma reactor combining transformer-coupled plasma (1st plasma chamber) with magnetic flux channel coupled plasmas (2nd plasma chambers) (based on [15]).

Choi [15,16] proposes a hybrid plasma reactor comprised of a ring-shaped transformer-coupled plasma with magnetic flux channel-coupled plasmas, to perform plasma processing for solids, powders, and gases. Figure 6 shows a sectional view of the hybrid plasma reactor. The transformer-coupled plasma is generated by delivering alternating current (AC) signals into the ring-shaped chamber (1st plasma chamber) by using a transformer, which is not illustrated in Figure 6. Although it is not clearly specified in the patent, both CCP and ICP can be generated in the first plasma chamber in this way. The hybrid plasma includes the magnetic flux channel-coupled plasmas. This configuration allows for a high control capability for plasma ion energy and a wide operation region from a low-pressure region to a high-pressure region. 

Vaduganathan et al. [17] study ozone generation by using a combination of AC surface discharge and pulsed DC corona discharge at atmospheric pressure. They find that a positive corona with surface discharge exhibits more efficient ozone generation than a negative corona. In both cases, the improvement of ozone generation can be attributed to a larger reaction volume. 

Dual cathode magnetron sputtering (DCMS) can also be regarded as a hybrid plasma. When two different metals are used for the targets, the synthesis of alloy films can be conveniently studied since each target can be operated independently. One example is the deposition of boron–carbon–nitride (BCN) films using DCMS [18], in the expectation that this will result in growth of cubic-BCN which has a structure like a diamond or cubic boron nitride. However, the results indicate that the introduction of boron is often difficult even with DCMS. DCMS can be used not only for alloy synthesis but also for improving the performance of magnetron sputtering. Specifically, by facing the magnetron targets with each other and arranging the configuration of the closed magnetic field in between, ion bombardment to the substrates and the growing film can be significantly improved [19]. 

**Figure 7:**
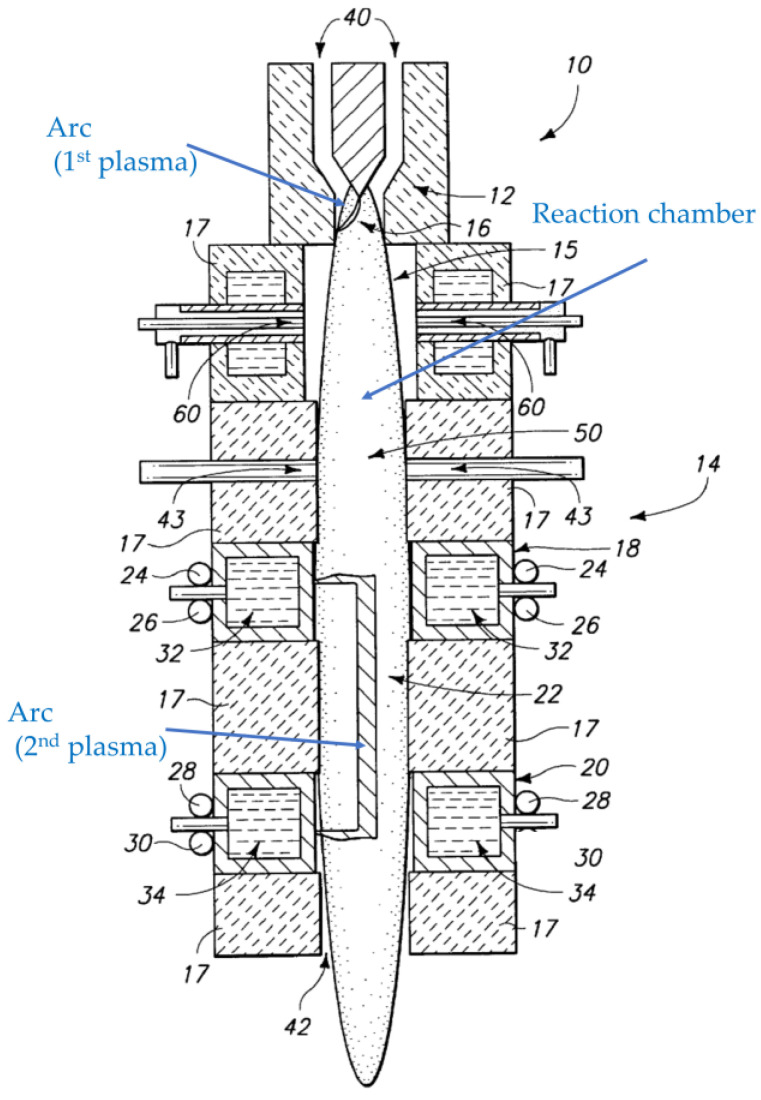
A cross-sectional view of a hybrid plasma reactor combining two arc plasmas (based on [20]).

**Figure 8:**
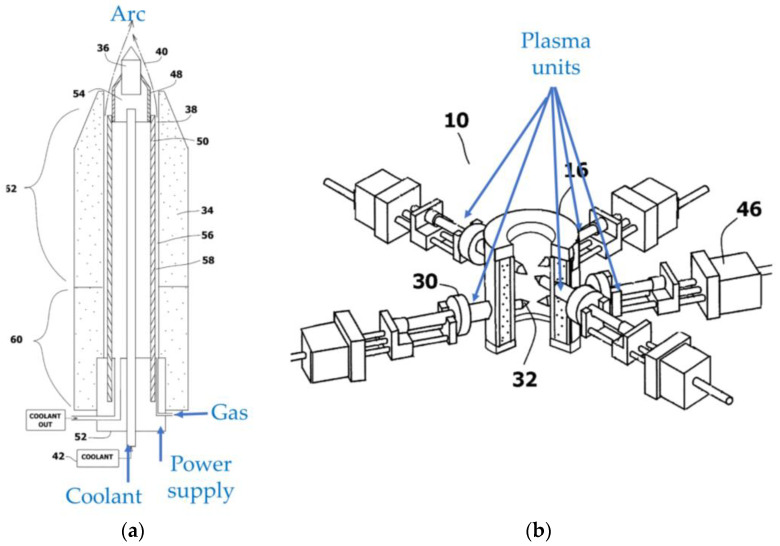
Schematic diagrams of a hybrid plasma source using a plurality of arc plasma sources. (**a**) A single plasma unit. (**b**) An assembled plasma source combining the plasma units (based on [21]).

Kong et al. [20] propose a hybrid plasma source combining two arc discharges, as shown in Figure 7. The first arc is used to ionize a hydrocarbon gas. The gas is subsequently fed to a second plasma source. Water is introduced between the arcs, and the second arc activates water to generate activated hydrogen and oxygen. The ionized hydrocarbon gas is reacted with the activated hydrogen and oxygen to generate synthetic gas. A plurality of arc sources can also be combined to produce synthetic gas [21], as shown in Figure 8. 

### 2.2. Ionized Magnetron Sputtering and Electron Cyclotron Resonance Magnetron Sputtering

**Figure 9:**
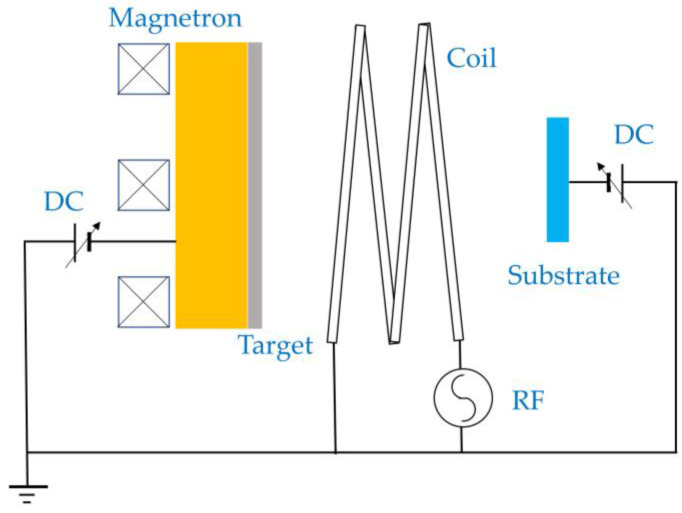
A schematic diagram of an ionized magnetron sputtering system.

One of the most successful uses of hybrid plasmas in this category from the late 1990s and early 2000s is in ionized magnetron sputtering (IMS) for thin film deposition, although it is never referred to as such. Magnetrons are used to sputter atoms against a target surface, and this technology has been praised as a step forward in sputtering technology [22]. However, the magnetron sputtering process can be improved further by increasing the ion bombardment flux to the film deposited on the substrate, by generating an ICP after the magnetron plasma to ionize the sputtered atoms [22]. Figure 9 shows a schematic diagram of a typical IMS setup. ICP is usually generated in a space between the magnetron and the substrate. If the substrate is biased, the ions can be accelerated toward the substrate so that a dense coating can be deposited, and the impact of collision of the ion to the film is greater as the potential difference is greater. 

Chen et al. [23] demonstrate improved crystallinity and low resistivity of tungsten films using an IMS. Chiu et al. [24,25] use IMS for stress control and texture formation of silver films, showing that the product of the ion flux and ion energy is the controlling parameter for texture formation. Tranchant et al. [26,27] present similar investigations to Chui et al. for MoCr films. Christou et al. [28] find that ionization of sputtered atoms strongly depends on the pressure of the sputtering gas. Schneider et al. [29,30] demonstrate low-temperature deposition of alumina using IMS. 

While efficient ionization and subsequent effects are demonstrated for metal depositions, carbon and carbon nitride films are also deposited using IMS in the application of hard and tribological coatings. Kusano et al. [31] find that positive substrate bias on the substrates showed higher sp and sp^2^-hybridized carbon contents than those without ICP and/or with negative bias voltages related to the selective etching of nitrogen and sp and sp^2^-hybridized carbon. The results are associated with the tribological properties of carbon nitride films [32]. The effects of using different inert gases, including helium, neon, and krypton, are also reported [33]. Angleraud et al. [34,35] also study the synthesis of carbon nitride films using IMS, finding selective areal deposition of carbon nitride on conductive and insulating parts of substrates. 

In a similar way, electron cyclotron resonance magnetron sputtering (ECR-MS) can also be considered a hybrid plasma. According to Yoshida [36] and Xu et al. [37], ECR-MS can be operated at 0.007 Pa. The electron cyclotron frequency and MW frequencies are synchronized to achieve resonance, hence the classification of ECR-MS as a hybrid plasma. It is noted that at higher pressures, collisions are pronounced to prevent efficient ECR acceleration. As a result, ECR-MS is most effective for pressures between 0.001 and 1 Pa [38]. When the pressure is higher than 0.1 Pa, wave damping by collisions is reported [39]. Therefore, ECR-MS is attractive for applications requiring low-pressure sputter depositions. 

IMS and ECR-MS are developed to enhance ionization of the plasma in the magnetron environment. This aim can also be achieved by supplying a DC-pulsed voltage of high energy density to the magnetron without hybridization. This technique is called high-power impulse magnetron sputtering (HIPIMS), also called high power pulsed magnetron sputtering (HPPMS) [40,41,42]. Due to the use of the pulsed high-voltage excitation, high ionization fraction of the sputtered species is expected. However, it is beyond the scope of this review, and hence a detailed description is not provided. 

### 2.3. Superposition

In Section 2.1 and Section 2.2, hybrid plasmas that are created by generating two or more plasmas simultaneously were discussed. However, processing using a single plasma can be improved by superposing different frequencies and/or waveforms of excitation voltages to generate and sustain a plasma. This type of plasma can also be regarded as a hybrid plasma. 

Ito et al. [43] report the synthesis of superconducting films by using a magnetron sputtering system, in which RF voltage is superimposed on a DC voltage. This sputtering method is called hybrid plasma sputtering or hybrid plasma magnetron sputtering. 

**Figure 10:**
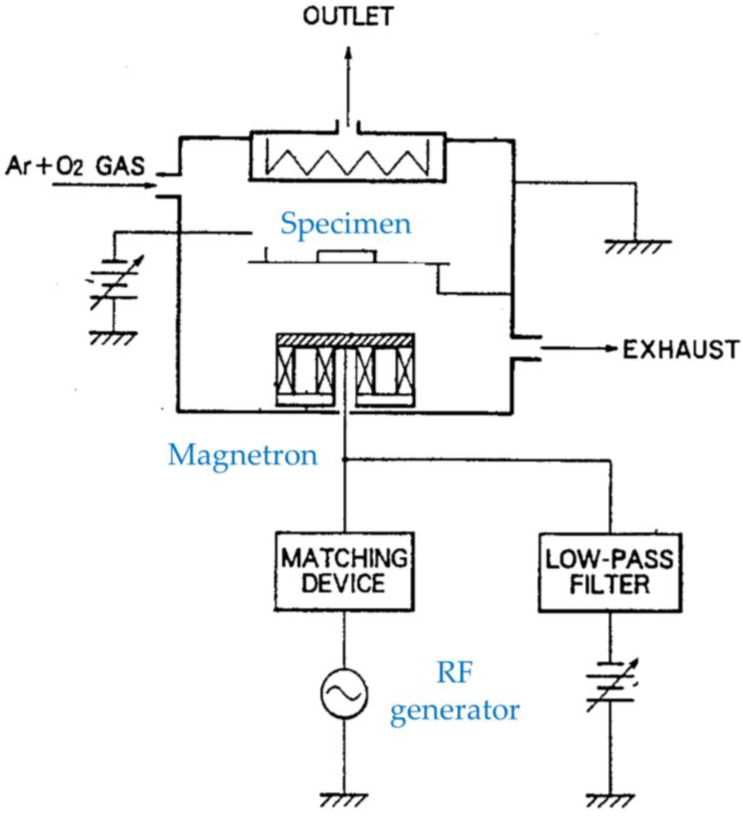
Hybrid plasma sputtering (based on [44]).

This technique enables the formation of superconducting films with high crystallinity due to the RF plasma, while achieving high-speed deposition due to the DC plasma. A setup of the hybrid plasma sputtering is illustrated in Figure 10, presented by Yoshida et al. [44]. 

It is reported that superposing different frequencies and/or waveforms of voltages is effective at producing ozone (for example [45]), similar to the pulsed excitation of the plasma [46]. Ahn et al. [45] combine AC-driven surface discharge with a pulsed DC corona discharge to demonstrate improved ozone production yield. 

It is noted that this type of technique is often referred to as “superimposition”. However, “superposition” is the proper technical term to be used.

## 3. Plasmas Driven by Multiple Electrical Sources Sequentially

One interpretation of hybrid plasmas is “the use of two plasma sources in the same vacuum chamber for two different applications” [47]. Alim et al. [47] refer to this as duplex surface treatment, and in their application, they utilize an ICP source and a magnetron cathode. The former is used to nitride and activate a given surface, while the latter is utilized for titanium deposition onto the surface. 

Duplex surface treatment is also used to improve tribological characteristics of metals; Díaz-Guillén et al. use pulsed plasma nitriding and a postoxidizing process to treat both hot work H13 tool steel [48] and cold work AISI D2 tool steel [49]. Researching such treatment for the latter material is especially important, considering its brittleness and wear rate; after the duplex surface treatment, the wear resistance is improved drastically because of the protective properties of the newly oxidized layer. 

It can be noted that hybrid plasmas are tools often used for surface strengthening. Treating Ti_6_Al_4_V with a combination of plasma immersion ion implantation with ion nitriding processes improves the hardness of this alloy considerably [50]. 

Another example is the deposition of Ti by magnetron sputtering followed by DLC coating using acetylene by hybrid plasma-activated metal–organic vapor deposition/physical vapor deposition (PA-MOCVD/PVD) [51] to synthesize hard coating for dental application. The technique has the disadvantage of using a single RF generator for the deposition of Ti and DLC with a risk of carbon contamination of the reaction chamber. 

**Figure 11:**
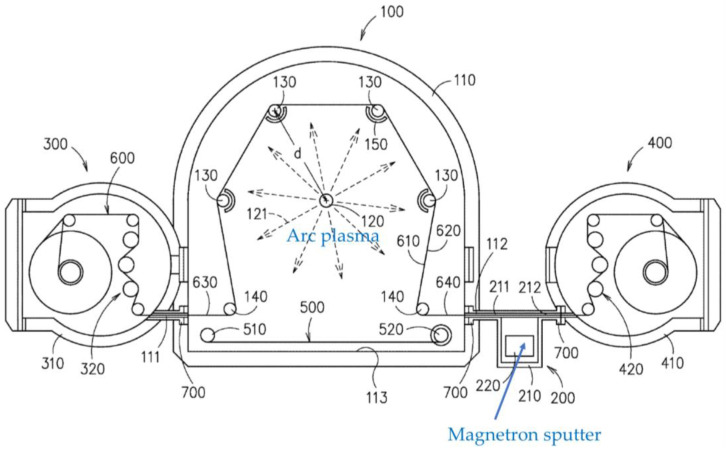
A schematic diagram of a roll-to-roll hybrid plasma modular coating system (based on [52]).

Hseih et al. [52] propose a roll-to-roll hybrid plasma modular coating system, which is comprised of at least one arc plasma processing unit and a magnetron sputtering unit, as shown in Figure 11. A web substrate is continuously fed to the arc plasma zone to pretreat the surface, and subsequently, a coating is deposited onto the activated surface by the magnetron sputtering. 

Although the above examples in this section are claimed as hybrid plasmas, they are most suitably regarded as two-step processes. 

## 4. Plasmas Having Properties of Thermal and Nonthermal Plasmas

Simultaneously achieving high chemical selectivity and efficient productivity is a challenge for atmospheric pressure plasma processing [3,4]. Nonthermal plasmas at a nonequilibrium state ensure elective chemical processes influence the chemical bonding of the molecules in the plasma and/or at the material surfaces to be treated. On the other hand, thermal plasmas, often characterized by high energy densities, enable efficient productivity. However, high nonequilibrium states with high energy densities are hard to achieve by general nonthermal plasmas or thermal plasmas. Therefore, the prospective of a plasma processing method that, by combining thermal and nonthermal plasmas possesses the advantages of both, is attractive. The gliding arc is one such type of plasma and is categorized as a hybrid plasma [1,53]. Note that while the gliding arc is technically a single plasma, the fact that it possesses the properties of both thermal and nonthermal plasmas is the reason that it is classified as hybrid. In this sense, perhaps it should be considered a separate kind of hybrid plasma. 

The gliding arc is shown to have many important applications, such as the cleaning of gas and controlling pollution, the conversion of fuel, and the production of hydrogen [1,53]. Gliding arcs also enhance combustion [54] and demonstrate sterilization effects [55]. Extensive research has been conducted on surface treatment for adhesion improvement using gliding arcs [56,57,58,59,60,61]. For example, wettability and adhesive strength of polyester composite plates with an adhesive (vinylester resin) are significantly improved, and as a result, the laminated structure of polyester composite and vinylester demonstrate significant fracture resistance [57,58]. The major treatment effect is attributed to the oxidation of the polyester [56,57,58,59,60,61]. 

Plasma diagnostics of the gliding arc have been extensively carried out and reported mainly by capturing photo images, using optical diagnostics and electric measurements. A typical gliding arc is a plasma column that is generated as an arc discharge and extends between two diverging electrodes in a turbulent gas flow. 

**Figure 12:**
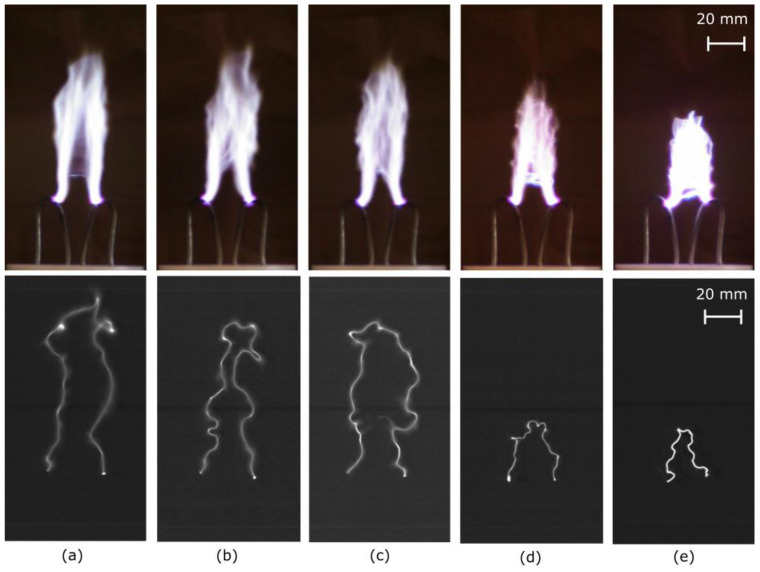
Photos images of the gliding arc I at air flowrates of: (**a**) 14 standard liter per minute (SLM), (**b**) 17.5 SLM, (**c**) 21 SLM, (**d**) 31.5 SLM, and (**e**) 42 SLM. The upper images: acquired by a normal camera using an automatic exposure time. The lower images: captured by a high-speed camera using an exposure time of 13.9 μs. Reproduced with permission from [62] Kusano et al., *Eur. Phys. J. D.*; published by Springer Nature, 2014.

**Figure 13:**
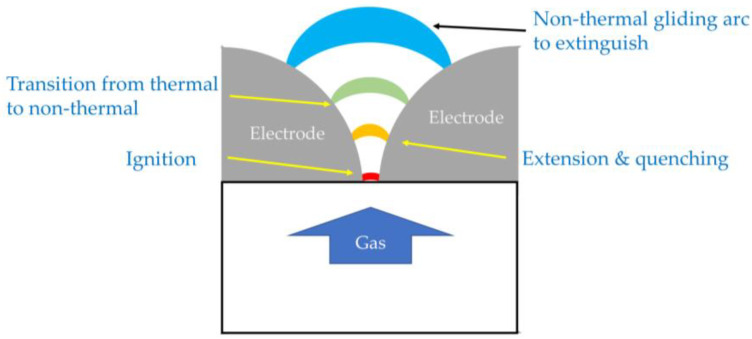
Gliding arc evolution.

The gliding arc is generally visualized as a thick photoemitting region. The upper images of Figure 12 show photos of AC-driven gliding arcs with different air flowrates [62]. However, true dynamic behaviors of gliding arcs are favorably observed by high-speed charged-coupled device (CCD) cameras with short exposure times [54,63,64,65,66,67,68,69,70,71,72]. Photos of gliding arcs observed by CCD cameras are shown in the lower images of Figure 12. The gliding arc is a thin plasma column. A typical evolution of a gliding arc is shown in Figure 13. The plasma column elongates until its extinction or until it encounters short-cutting events. 

Voltage-current (VI) characteristics are often researched since their measurement gives direct information on ignition, short-cutting events, power consumption, evolution, and fluctuation of gliding arcs [63,64,65,66,67,69,70,72,73,74,75,76,77,78,79,80]. 

Optical emission spectroscopy (OES) is widely used for plasma diagnostics. It is regarded as an ideal noninvasive technique that does not contaminate the plasma environment. OES of gliding arcs is reported for studying reactive species in the plasma such as excited-state OH radicals and NO radicals, as well as estimating rotational and vibrational temperatures of gliding arcs [71,73,74,81,82,83,84,85]. It is noted that OH radicals are known to be highly oxidative agents, and that photoemission of OH radicals is detected even 60 mm away from the edge of the electrodes [56]. The result indicates that the gliding arc can be advantageously applied for surface modification of 3D bulky objects for adhesion improvements [56]. 

**Figure 14:**
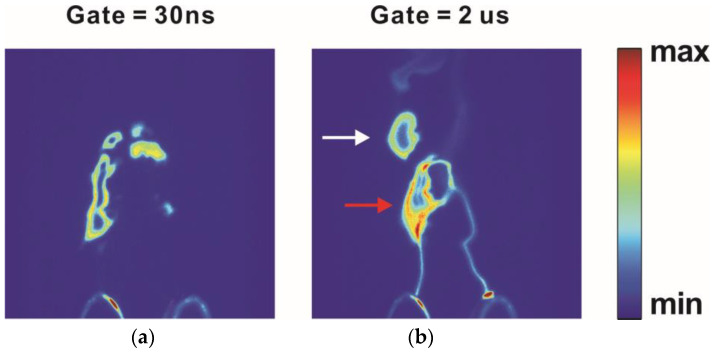
LIF images captured with two gate times of (**a**) 30 ns (LIF signal only) and (**b**) 2 μs (both LIF signal and plasma column emission). Both the red and white arrow in (**b**) indicate ground state OH. Adapted with permission from [67] © The Optical Society.

Laser-induced fluorescence (LIF) can be employed to measure distribution of the ground-state OH, which does not emit photons by itself. Figure 14 exemplifies LIF images at the vicinity of a gliding arc [67]. The detected ground-state OH exhibits a hollow structure around the plasma column. It is therefore indicated that the ground-state OH can be generated by the decay of the excited OH that is detected in the discharge column by OES.

In addition to the observations and characterizations of gliding arcs by plasma diagnostics, theoretical studies are reported in terms of energy balance [1,53,86,87,88,89,90,91] and force balance [66,92]. Furthermore, equivalent electric circuits are proposed for studying gliding arcs [1,53,62]. The plasma string model [89,90,91] is regarded as an energy balance equation of the equilibrium gliding arc [1,53], taking a small part of the plasma column and neglecting radial convection or turbulent effects. The Elenbass–Heller equation is regarded as a simplified governing equation of a gliding arc [93], which can accurately describe the electrical field, electrical conductivity, temperature, and plasma column radius of the gliding arc. 

It is desirable for many industrial applications that a gliding arc does not extinguish in a short time after ignition and is sustained as the nonthermal gliding arc. This is so that the energy introduced to the gliding arc can be used efficiently. Otherwise, a significant amount of energy is consumed for reignition. Therefore, studying stability, fluctuations, and thermal to nonthermal transition of gliding arcs is important for better controlling the gliding arcs. Ref. [62] reports an analytical calculation based on Ohm’s law for studying the critical length of AC gliding arc discharge columns. The study indicates that the critical length of the AC gliding arc can be larger than that of a DC gliding arc. The analysis further shows that the critical length can be increased by increasing the AC frequency for driving the gliding arc, decreasing the serial resistance connected to the gliding arc electrode, and reducing the gas flowrate (Figure 12). The predicted dependence of air flowrate on the length of the gliding arc is experimentally demonstrated in [62]. 

## 5. Plasmas Enhanced by Additional Energy

### 5.1. Combination with Thermal Energy

Plasmas whose performances are enhanced by additional energy are also frequently referred to as hybrid plasmas. 

Welding is a process that benefits from the use of these hybrid plasmas. The combination of a concentrated plasma arc and a “metal active gas” (MAG) has been found to be a more favorable replacement to traditional welding methods, since it is more efficient, has less weld metal content, and requires less preparation of welded metal joints [94]. Hybrid plasma arc welding has also been seen to reduce the weld width by 25–50% compared to the more traditional plasma arc welding, while keeping the energy output the same [95]. 

Furthermore, the combination of a hybrid plasma arc and micro rolling (HPAMR) technology have been used to coat Inconel 718 superalloy, often used in areas such as aviation. Due to the rolling process, this combination leads to an improvement in wall morphology of the coating, refinement of internal grains, elimination of defects and microporosity, an increase in the precipitation of the strengthening phase, and an improvement of the mechanical properties, much more so than plasma arc additive manufacturing [96]. 

Plasma MIG, which is hybrid plasma arc welding with gas–metal arc welding in a single torch, is more effective than the traditional pulsed MIG process since plasma can preheat and stabilize the MIG arc [97]. 

Another example of combining plasma with heat is the amalgamation of dielectric barrier discharge (DBD) plasma with external heating, resulting in a two-stage hybrid plasma–thermal system (HPTS) that shows promise in converting methane to ethylene and hydrogen [98].

### 5.2. Combination with Electron Beam

Hybrid plasma of this nature can also be applied in other ways, such as in the realm of medicine and biology. Upon exposure to oxygen hybrid plasma with the assistance of electron beams (EB), it is found that the hydrophilic tendencies of thin chitosan films are greatly improved for a period of nearly 50 days [99]. Additionally, it is noted that hybrid plasma can be used as a reliable bactericide and sterilizer, in comparison to oxygen RF plasma [99]. In this research, the main ionizer was an EB, while the other ionization source was an RF discharge [99]. 

**Figure 15:**
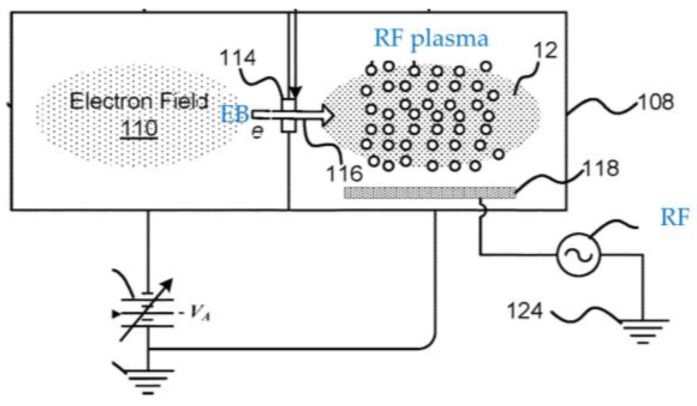
A schematic diagram of a hybrid plasma setup combining an EB source with RF plasma (based on [100]).

Chen et al. [100] also combine an EB source with RF plasma, as shown in Figure 15. The introduction of EB assists ignition and sustainment of the plasma, as well as controlling the plasma properties. Laser-accelerated EB can also be used to power a plasma accelerator, which makes studies with such devices far easier at commonly available high-power laser facilities [101]. 

### 5.3. Combination with Photoexcitation

**Figure 16:**
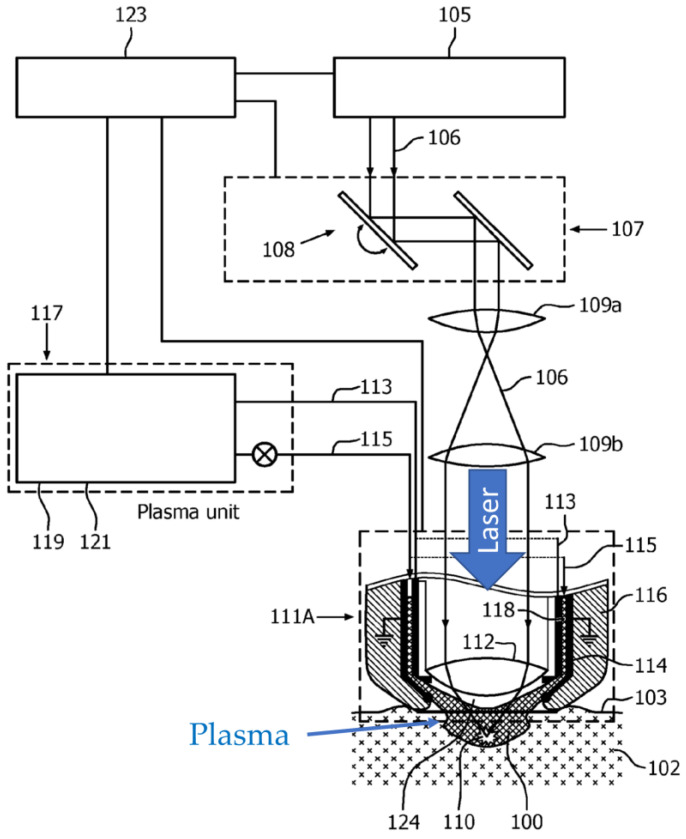
A schematic diagram of laser-activated atmospheric pressure plasma (based on [102]).

Plasma processing can also be further activated by photoexcitation, for which laser sources are commonly used. Varghese et al. [102] propose a skin treatment device using an atmospheric pressure plasma, to which a laser beam is irradiated to facilitate the plasma treatment, as shown in Figure 16. 

**Figure 17:**
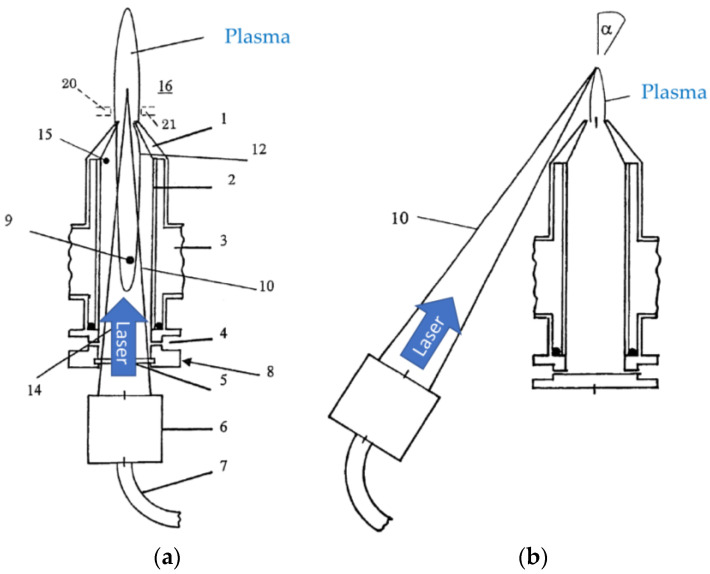
Schematic diagrams of laser-activated atmospheric pressure MW plasma. (**a**) The directions of the MW plasma and the laser beam are coaxially aligned. (**b**) The laser beam is directed at an angle to the MW plasma (based on [103]).

Bayer et al. [103] combine MW plasma with laser irradiation for welding application, as shown in Figure 17. There are two configurations presented. One is similar to [102] where the directions of the MW plasma and the laser beam are coaxially aligned (Figure 17a). Another configuration is that the laser beam is directed at an angle to the MW plasma (Figure 17b). It is functionally the same as Figure 17a, but the advantage of this configuration is that the parts can be produced separately. 

### 5.4. Combination with Acoustic Energy or Mechanical Vibration

**Figure 18:**
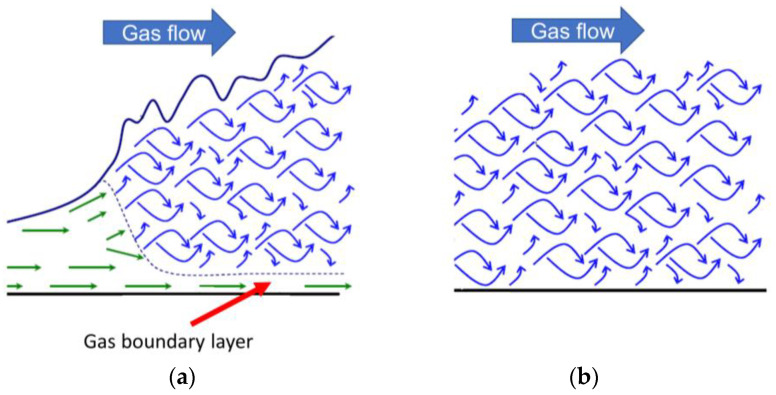
A gas boundary layer (green) (**a**) without ultrasound and (**b**) with ultrasound (based on [112]).

During atmospheric pressure plasma processing, a process gas is usually fed to the plasma. However, even when the gas flow is fully turbulent, a gas boundary layer sticks at the surface of the material that is to be treated with plasma. Plasma reactive species such as ions, electrons, high-energy neutrals, and radicals diffuse through the gas boundary layer and activate and/or react with the material surface. Since these reactive species generally have short lifetimes, only a small fraction of them can reach the material surface. It is reported that ultrasonic waves with a sound power level (SPL) above approximately 140 dB can reduce the thickness of the gas boundary layer and that the pressure plasma treatment can be made highly efficient by simultaneously irradiating ultrasonic waves to the treating surface [104,105,106,107,108,109,110,111,112]. This is because the acoustic energy can be delivered efficiently at high gas pressures, which reduces the thickness of the gas boundary layer, as shown in Figure 18. 

As a result, the probability of the plasma reactive species reaching the surface before inactivation is higher when ultrasonic waves are applied, which improves surface modification efficiency. Combinations of a plasma and ultrasonic waves are investigated for understanding the interaction between plasma and acoustic waves [113,114,115], electrical discharge machining [116,117], plasma etching [118], ozone production [119,120,121,122], decomposition of volatile organic compounds (VOC) [123], charging performance improvement of corona chargers [124], and surface modification [104,105,106,107,108,109,110,111]. 

**Figure 19:**
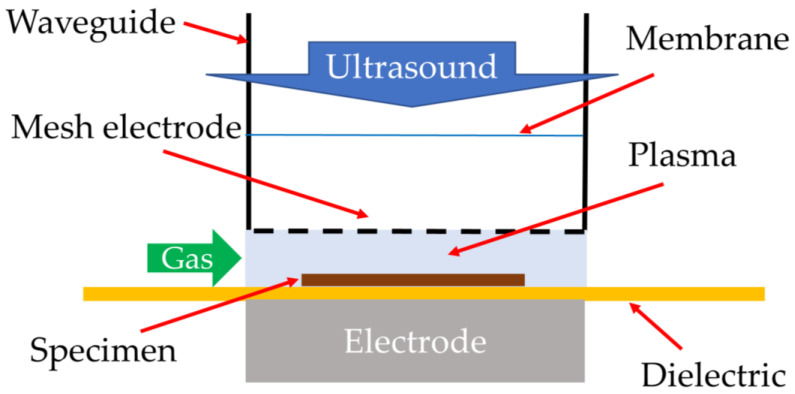
A schematic diagram of ultrasound-enhanced plasma.

Ultrasonic-assisted electrical discharge machining combines ultrasonic waves with plasma using a solid-state electroacoustic transducer to vibrate material surfaces. However, due to the significant acoustic impedance mismatch between a solid material and a gas, most of the acoustic power generated by a solid-state transducer cannot be efficiently transmitted into the surrounding gas. Therefore, the thickness of the gas boundary layer at a material surface cannot be reduced efficiently. On the other hand, a gas jet ultrasonic generator excites acoustic waves in a gas and is suitable to eliminate or reduce the gas boundary layer. Figure 19 exemplifies a setup of an ultrasound-enhanced hybrid plasma. Ultrasonic waves are introduced to the plasma volume using a waveguide. A membrane can be used to separate the plasma gas from the ambient air. A mesh electrode is used as the upper electrode so that the ultrasound can pass it without a significant loss of acoustic energy. In this example, a gas jet ultrasonic generator is used.

Plasma etching can also be enhanced with ultrasonic vibrations. Here, the surface to be treated is vibrated mechanically. These vibrations increase the plasma collision energy since the vertical vibration in the plasma leads to a relative speed enhancement of the particles in the plasma. The incident angle of particles and the energy at which they collide are also increased. The increase in collision angle leads to better anisotropy in plasma etching, while the energy increase results in an improvement of the etch rate [118]. 

## 6. Plasmas Operated in a Unique Medium

There is a growing interest in operating plasmas in different mediums, expecting new results for material synthesis, processing, and reaction fields. Examples of such plasmas include plasmas generated in a packed catalyst, plasmas generated in a liquid, and plasmas generated in a supercritical fluid. 

The study of plasma catalysis has recently been growing in popularity; this involves inserting a catalyst into a plasma volume, in expectation of a synergistic reaction resulting from catalytic activity and the use of plasma [125,126]. Methanation, methane coupling, and CH_4_ reforming, as well as dry reforming of methane, can all be performed using plasma catalysis [125,127,128]. The same also applies to CO oxidation and CO_2_ decomposition [125]. Other processes that can be executed using plasma catalysis are the decomposition of nitrogen oxides and the synthesis of H_2_O_2_ [125,126]. Another application is in Fischer–Tropsch synthesis, which is the conversion of CO_2_, CH_4_, and waste biomass to more useful chemical substances and fuels [125]. Plasma catalysis can also be used in the removal of pollutants, such as volatile organic compounds (VOC) consisting of aromatics, alcohols, ketones, and esters, sterilization of environments and wastewater, as well as the degradation and removal of pesticide residues [125,126]. For a more comprehensive guide to plasma catalysis, readers are recommended to refer to [125,126]. 

Another quickly developing field of research concerns plasma discharges formed in liquids, as well as plasma discharges interfacing with liquids [129]. The advantage of liquid discharge plasmas is that they can achieve high-speed oxidation in water due to it emitting photons and chemical compounds with very strong oxidizing power [130,131,132]. More specifically, the OH radicals produced by this kind of plasma have a higher energy than those produced by UV light or ozone [130,131,132]. These plasmas offer “unique conditions”, such that they can be used to decontaminate pathogens, synthesize nanostructures, and treat contaminated liquids such as water [129]. For example, low-temperature corona plasma can be employed for wastewater treatment, either in a solution, or alternatively through direct barrier discharge plasma [130]. It seems that such plasmas also have consequences that are beneficial for the environment. Liquid-phase plasma, in conjunction with a photocatalyst, can be used to decompose hydrocarbons into hydrogen and carbon, without generating carbon dioxide as a byproduct [130]. For a more exhaustive and thorough review of liquid phase plasmas, readers are recommended to refer to [129]. 

**Figure 20:**
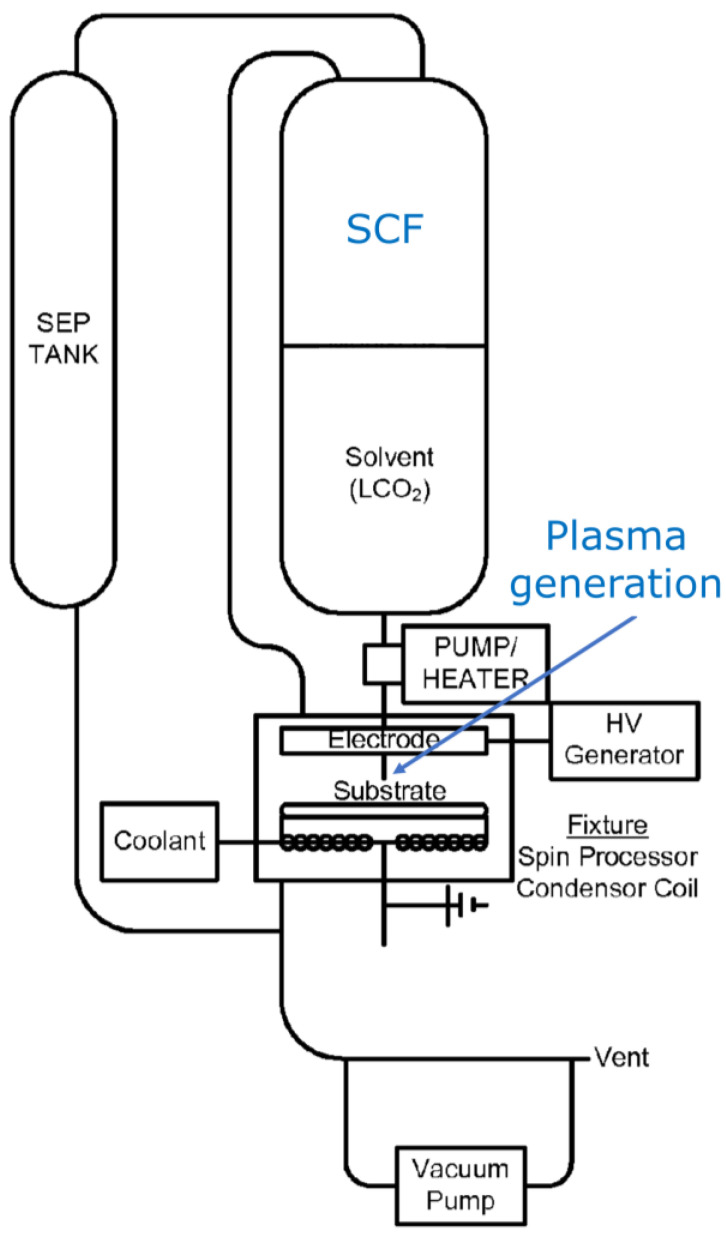
A schematic diagram of an SCF chamber with a plasma generation setup (based on [139]).

It is anticipated that subjecting a plasma to a supercritical fluid (SCF) may result in unique characteristics and reactions that differ from those of a normal gaseous state plasma [133]. Studies reported of plasmas in SCFs [134] include plasma ignition, plasma diagnostics, decomposition, synthesis [135], and deposition. However, the fundamental characteristics of plasmas in SCFs are still not understood. In an SCF, the plasma may not be controlled sufficiently at a nonequilibrium state or may be substantially unevenly distributed, and it follows that the plasma density is not high enough. There is a risk of generating a thermal or arc plasma [136,137,138], losing chemical selectivity. High voltage is required to generate a plasma in an SCF, and the gap between the electrode should be small enough for igniting and sustaining a plasma in an SCF environment. Jackson [138] presents generation of a plasma in an SCF, called “super-atmospheric plasma”, to enhance surface processing. Figure 20 exemplifies the configuration of the setup, comprised of SCF and plasma [139]. 

## 7. Characterization Techniques

For a deep understanding of hybrid plasmas and their performances, a variety of characterization techniques are necessary. This chapter briefly lists important characterization techniques for plasma diagnostics as well as applications of hybrid plasmas.

Plasma diagnostic techniques include invasive methods like inserting probes in a plasma, passive spectroscopies such as OES [140], active spectroscopies such as LIF [66], Fourier transform infrared (FTIR) spectroscopy [141], mass spectroscopy [142], and electrical measurements. For a comprehensive review of plasma diagnostics, see [143,144]. 

For the applications of plasma processing, a broad range of material characterizations is needed. There are no limitations for the choice of characterization techniques. Characterization techniques include, but are not limited to: microscopic analysis, spectroscopic analysis, structural analysis (molecular structure, molecular weight, crystallinity, defects), elemental and compositional analysis, and measurement of chemical or physical (mechanical, thermal, electrical etc.) properties. Some of the fundamental characterization techniques are described in [145]. Major areas of the applications include characterization of gas, liquids, solid materials, and materials surfaces. Among them, surface characterization plays a unique and important role in relation to hybrid plasma processing, since in many applications, the essential changes after the plasma processing can be only at the surfaces or their vicinities. Commonly used surface characterization techniques include contact angle measurement for the estimation of surface tensions [146,147,148], FTIR spectroscopy, X-ray photoelectron spectroscopy (XPS), energy-dispersive X-ray spectrometry (EDS), Time of Flight secondary ionized mass spectrometer (TOF-SIMS) for surface chemistry analyses, atomic force microscopy (AFM), optical microscopy, and scanning electron microscopy (SEM) for morphological analyses [4]. Contact angle measurement is widely used in the industry. It is the simplest method for surface characterization, sensitively detecting the surface property with the analysis depth of approximately 1 nm or less. 

## 8. Discussion

In this review, different types of hybrid plasmas have been explained and discussed. While the ambiguity of the term “hybrid plasmas” leads to a large variety of types of enhanced plasma, one must also consider whether a given “hybrid plasma” can suitably be called “hybrid” in terms of its configuration and effects; for example, one must contemplate whether a two-step process can appropriately be coined as a “hybrid” process. Additionally, while different configurations of plasmas may be interesting to discuss and file patents for, discussions must be had regarding what kinds of advantages these have in comparison to their nonhybrid counterparts. Without this, a hybrid–plasma configuration for the sake of discovering a new configuration may prove to be without much meaning.

**Figure 21:**
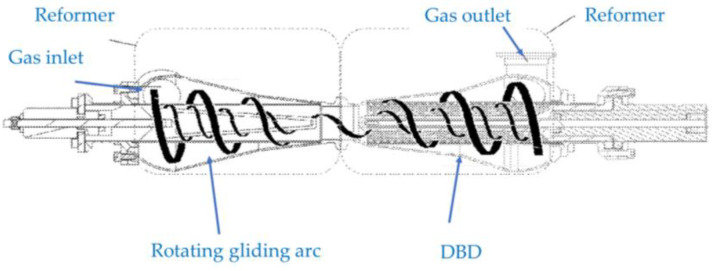
A cross-sectional view of a gas reformer combining a gliding arc and a DBD (based on [149]).

How can research then move forward in this field of study? One idea worth mentioning is combining hybrid plasmas. Hill et al. [149] propose a gas reformer by a further hybridization process, comprised of an upstream gliding arc and downstream DBD in combination as shown in Figure 21. It is a combination of the concepts from Section 2.1 and Section 4. 

**Figure 22:**
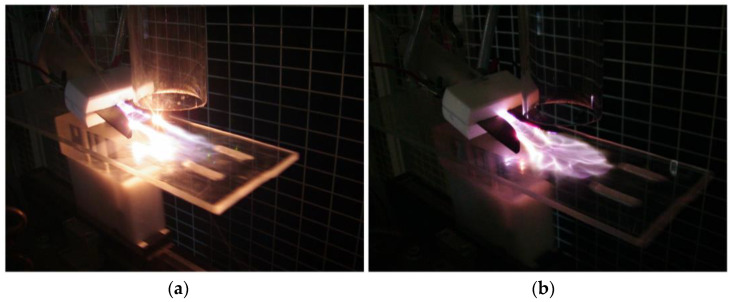
Photo images of the gliding arc (**a**) without ultrasonic irradiation, and (**b**) with ultrasonic irradiation. Reproduced with permission from [109] Kusano et al., *Surf. Eng.*; published by Taylor & Francis, 2012.

In a similar yet slightly different way, Kusano et al. report a process to combine gliding arc and ultrasonic irradiation [109,150,151] as shown in Figure 22. The gliding arc is tilted, and ultrasound is irradiated vertically, relative to the specimen holder. The combination of gliding arc with ultrasonic irradiation further improves the treatment effect. 

It is also worth mentioning that even though the gliding arc itself is already a hybrid plasma, it takes the form of a single plasma, and therefore it can easily be subjected to further hybridization. 

The assumption in studying hybrid plasmas is that treatments or processing by them are more beneficial compared to their nonhybrid counterparts. However, one must also discuss the disadvantages that accompany hybrid plasmas. For example, if the configuration of the hybrid plasma is relatively complicated, it can result in the need to produce expensive equipment. One must also consider the increase in the cost of manufacturing, maintenance, and investment for such a device. There are also environmental impacts to be considered, for the activation of hybrid plasmas may lead to the production of substances toxic to health or to the environment. Here, major environmental impacts of using plasma processing are reported to be due to the use of electricity for the generation and operation of the plasma [152]. It is therefore suggested that one be very selective in choosing which configurations of hybrid plasmas are industrially useful. Specifically, the advantages and disadvantages of each configuration must be weighed very carefully, especially when the effects of improvements by hybridization are not significant. 

Taking an example in plasmas combined with ultrasonic irradiation, oftentimes this configuration on its own only yields moderate improvements [108,109,110,111]. However, under certain conditions, the improvements can be much more noticeable. It has been observed that even with under a 5-min exposure in helium DBD treatment at less than 1 W, the water contact angle decreases much more significantly with added ultrasonic irradiation than without, and this is especially visible for lower frequencies of ultrasound [61,107]. It has also been observed that if PET films are treated by DBD plasma without ultrasonic irradiation, it takes 30 times longer to reach the same level of plasma treatment effect compared to when ultrasonic irradiation is applied [104]. These results make a strong case for further pursuing this type of hybrid plasma. 

Consider also that the application of hybrid plasma may also result in the obtainment of results that are simply unachievable by ordinary nonhybrid plasma, regardless of the energy and time used to operate it. These types of improvements are also very important to examine. For example, it is known that a DBD in air is an assembly of filamentary micro-discharges [153] and material surfaces that are exposed to it are inevitably unevenly treated. On the other hand, when the DBD–ultrasound configuration is operated in air at atmospheric pressure, polymer surfaces are treated uniformly [110]. It is foreseen that when air DBD is used and uniform treatment is required in bio or semiconductor applications, the ultrasound-assisted hybrid plasma processing can be worth consideration.

## 9. Summary and Outlook

Various types of hybrid plasmas are reviewed, referring to scientific literature and patents. They are briefly summarized below. 

Plasmas driven by multiple electrical sources simultaneously (Section 2) are presented by combining two plasmas (Section 2.1), magnetron sputtering with RF or ECR plasma (Section 2.2), and plasma generated by superposing different frequencies or waveforms of voltages (Section 2.3). A noticeable uniqueness of the plasmas in Section 2.3 is that it is a single plasma, while those of Section 2.1 and Section 2.2 are a combination of a plurality of plasmas. 

Plasmas driven by multiple different electrical sources sequentially (Section 3) are regarded as two-step processes rather than hybrid plasmas.

Plasmas having properties of thermal and nonthermal plasmas (Section 4) are represented by gliding arcs. They are also single plasmas but are generated without superposing excitation voltages. 

Plasmas enhanced by additional energy (Section 5) are demonstrated by combining plasmas with thermal energy (Section 5.1), electron beam (Section 5.2), photoexcitation (Section 5.3), and acoustic energy or mechanical vibration (Section 5.4). The type of additional energy can be adequately selected in accordance with specific applications. 

Plasmas operated in a unique medium (Section 6) are relatively new, and unique characteristics of reaction fields for chemical processes can be expected for the realization of novel materials processing. 

Characterization techniques are listed in Section 7, and scopes of hybrid plasmas are discussed in Section 8. 

There is no simple solution or criteria to select for the best choice of processes. However, appropriate selections must be made for the benefit of research and industry. The above examples will hopefully give the reader some insight into hybrid plasmas and encourage them to make further discoveries in this field of research. 

## Data Availability

No new data were created or analyzed in this study. Data sharing is not applicable to this article.
